# Screening for Fetal Aneuploidy and Sex Chromosomal Anomalies in a Pregnant Woman With Mosaicism for Turner Syndrome—Applications and Advantages of Cell-Based NIPT

**DOI:** 10.3389/fgene.2021.741752

**Published:** 2021-09-14

**Authors:** Line Dahl Jeppesen, Lotte Hatt, Ripudaman Singh, Palle Schelde, Lotte Andreasen, Sara Markholt, Dorte L. Lildballe, Ida Vogel

**Affiliations:** ^1^ARCEDI Biotech, Vejle, Denmark; ^2^Department of Clinical Medicine, Center for Fetal Diagnostics, Aarhus University, Aarhus, Denmark; ^3^Department of Clinical Genetics, Aarhus University Hospital, Aarhus, Denmark; ^4^Department of Molecular Medicine (MOMA), Aarhus University Hospital, Aarhus, Denmark

**Keywords:** cell-based noninvasive prenatal testing, sex chromosomal aneuploidies, turner mosaic, non-invasive prenatal testing, extravillous trophoblast

## Abstract

**Background:** Cell-free NIPT and cell-based NIPT are risk-free testing options using maternal blood samples to screen for fetal aneuploidies, but the methods differ. For cell-free NIPT, the fetal fraction of cell-free DNA in plasma is analyzed with a high background of maternal DNA. In contrast, for cell-based NIPT, a limited number of the rare, intact fetal cells are isolated for the genetic analysis. This case demonstrates the differences regarding testing for fetal sex-chromosomes anomalies (SCAs) between these two tests.

**Materials and Methods:** A pregnant woman with mosaicism for Turner syndrome opted for NIPT in first trimester. For the cell-free NIPT analysis, DNA extraction, genome-wide massive parallel sequencing, and data analysis were carried out as described by the kit manufacturer (Illumina©, San Diego, CA, USA). For cell-based NIPT, the first sample gave no result, but the woman consented to repeat cell-based NIPT. After whole genome amplification and STR analysis, fetal DNA from three individual fetal cells was subjected to chromosomal microarray (aCGH, Agilent oligoarray, 180 kb).

**Results:** Fetal fraction was 7%, and cell-free NIPT showed 2 copies of chromosomes 13, 18, and 21 and a decreased proportion of chromosome X, suggestive of fetal Turner syndrome. In contrast, the cell-based NIPT result showed no aneuploidy and two X-chromosomes in the fetus.

**Conclusion:** cell-based NIPT may provide a non-invasive testing option to screen for SCAs in women with mosaicism for monosomy-X in blood, where cell-free NIPT cannot discriminate whether the X-loss is maternal or fetal.

## Introduction

Cell-free non-invasive prenatal testing (cfNIPT) through analysis of cell-free fetal DNA (cffDNA) in maternal plasma is widely implemented as a screening for fetal trisomy 13, 18, and 21, and often also sex chromosomal aneuploidies (SCAs) (Gadsbøll et al., 2020). cfNIPT is highly sensitive and specific for trisomies 21 (sensitivity: >99%), 13 (sensitivity: >98%), and 18 (sensitivity: >99%) (Rose et al., 2020), but the concordance for SCAs is lower (from 90.5 to 100%) (Pertile et al., 2021). Maternal SCAs is a known cause of false positive cfNIPT results, which may lead to redundant referral for invasive testing (Wang et al., 2015; Zhang et al., 2020).

SCAs is a group of genetic conditions characterized by numeric abnormalities of sex chromosomes and includes 45,X (Turner syndrome), 47,XXY (Klinefelter syndrome), 47,XXX and 47,XYY, that affects 1/400 newborns, collectively being more common than Downs syndrome (Nielsen and Wohlert, 1991). Monosomy X, clinically diagnosed as Turner syndrome, is the most common SCA in female conceptions, and in adulthood the condition may occur in three different groups of women. In the non-mosaic state, the first group, Turner syndrome will develop. These women have general health complications (Gravholt et al., 2019) and are typically infertile. Women with mosaicism for Turner syndrome, the second group, may present the full phenotypic range—from manifestation of Turner syndrome to being completely without phenotypic traits of Turner syndrome. Some of these women are unaware of their condition, and others are diagnosed with mosaic Turner syndrome in childhood or when trying to conceive. This group of women may have fertility problems, but some conceive. Their risk of fetal aneuploidy is only documented in smaller case series but seems to be elevated for trisomy 21 and sex chromosome aberrations (Birkebaek et al., 2002; Sybert, 2002; Bernard et al., 2016). Therefore, in Denmark, women with mosaicism for Turner syndrome are offered invasive testing in pregnancy but may wish for risk-free non-invasive testing opportunities as obtained pregnancies may be rare in these women. The third, and most common group, consists of women with an age-dependent loss of X chromosome in blood (Guttenbach et al., 1995; Russell et al., 2007). Testing-wise these women will be indistinguishable from women with mosaicism for Turner syndrome, and likely their risk of aneuploidy in pregnancy will also be higher due to advanced maternal age. These women will also be represented among women opting for NIPT.

In this report, we demonstrate how cell-based NIPT (cbNIPT), a developing technology based on isolation of fetal extravillous trophoblasts (fEVTs) from maternal blood, provides a non-invasive screening option for both aneuploidies and sex chromosome aberrations to a woman with Turner mosaicism. cbNIPT has, in a research setting, been an option for all women opting for cfNIPT in the Central Denmark region since 2018.

## Case Presentation

A woman under the age of 30 was diagnosed with mosaicism for Turner syndrome after 3 spontaneous miscarriages. She had monosomy X in 27% of examined metaphases in peripheral blood and her karyotype was mos 45,X[4]/46,XX[6].ish mos X(DXZ1x1)[12]/X(DXZ1x2)[38]. The woman had no clinical signs of Turner syndrome. Shortly after diagnosing her with mosaicism for Turner syndrome, she became pregnant without reproductive assistance. She was counseled on an option of either invasive vs. non-invasive testing (cfNIPT) according to Danish guidelines and at gestational age (GA) 10+3, she opted for NIPT prior to her combined first trimester screening. In the Central Denmark Region, women opting for cfNIPT are asked if they additionally want the cbNIPT carried out in a research setting and written informed consent for cbNIPT was obtained. From the first cbNIPT sample, one fEVT contaminated with maternal DNA was sampled, and thus, no further analysis was conducted. The woman attended the first trimester screening at the Department of Obstetrics and Gynecology at a Regional Hospital in Denmark and received a normal risk estimate for Downs syndrome and the nuchal translucency was normal (2.7 mm) and normal biochemistry. The woman chose resampling of blood for a second run of cbNIPT when a reanalysis was offered at GA 13+1.

The woman did not opt for invasive prenatal diagnosis after NIPT results.

## Methods

### Cell-Free NIPT

Cell-free NIPT was performed at the Department of Clinical Genetics, Aarhus University Hospital. For cfNIPT analysis, 20 mL blood was sampled in Cell-Free DNA BCT tubes (Streck laboratories, USA) at GA 10+3 and cell-free DNA extraction from plasma, genome-wide massive parallel sequencing, and data analysis were conducted following the kit manufacturer's instructions (Illumina©, San Diego, CA, USA) for TG TruSeq® Nano DNA Sample Preparation kit v1.0 + TG NSQ 500/550 High Output Kit v2. VeriSeq NIPT Analysis Software v1 was used for analysis of the fetal fraction and aneuploidy status.

### Cell-Based NIPT

For cbNIPT, 30 mL of peripheral blood was first collected at GA 10+3, and at repeat analysis at GA 13+1, 60 mL blood was sampled and split into 2 × 30 mL samples in accordance with the protocol. Blood was collected in Cell-Free DNA BCT tubes (Streck laboratories, USA). Fetal extravillous trophoblasts (fEVTs) were enriched as previously described using Magnetic Activated Cell Sorting (Miltenyi Biotech, Germany) (Kølvraa et al., 2016). fEVT candidates were isolated using BD FACSMelody™ Cell Sorter (BD Biosciences, US) for single-cell sorting. Whole genome amplification (WGA) was performed using Smart Picoplex WGA (Takara, Japan). fEVTs were validated to be of fetal origin and not contaminated with maternal DNA by short tandem repeat (STR) analysis using GlobalFiler™ PCR Amplification Kit (Thermo Fisher Scientific, US) following manufacturer's instructions. Fragment size separation by capillary electrophoresis was run on an Applied Biosystems 3500 Genetic Analyzer instrument and data analysis was conducted using GeneMapper ID-X software (Thermo Fisher Scientific, US). Copy number variation (CNV) by array Comparative Genomic Hybridization (aCGH) analysis was performed on single-cell WGA from fEVTs using SurePrintG3Human CGH 4 × 180K arrays from Agilent Technologies. Reference DNA for the fEVT WGA product was a pool of 10 WGA products from lymphoblastic genomic DNA (Promega, USA). CNVs were detected using the adm-2 algorithm with a 5 Mb threshold.

## Results

**Figure 1:**
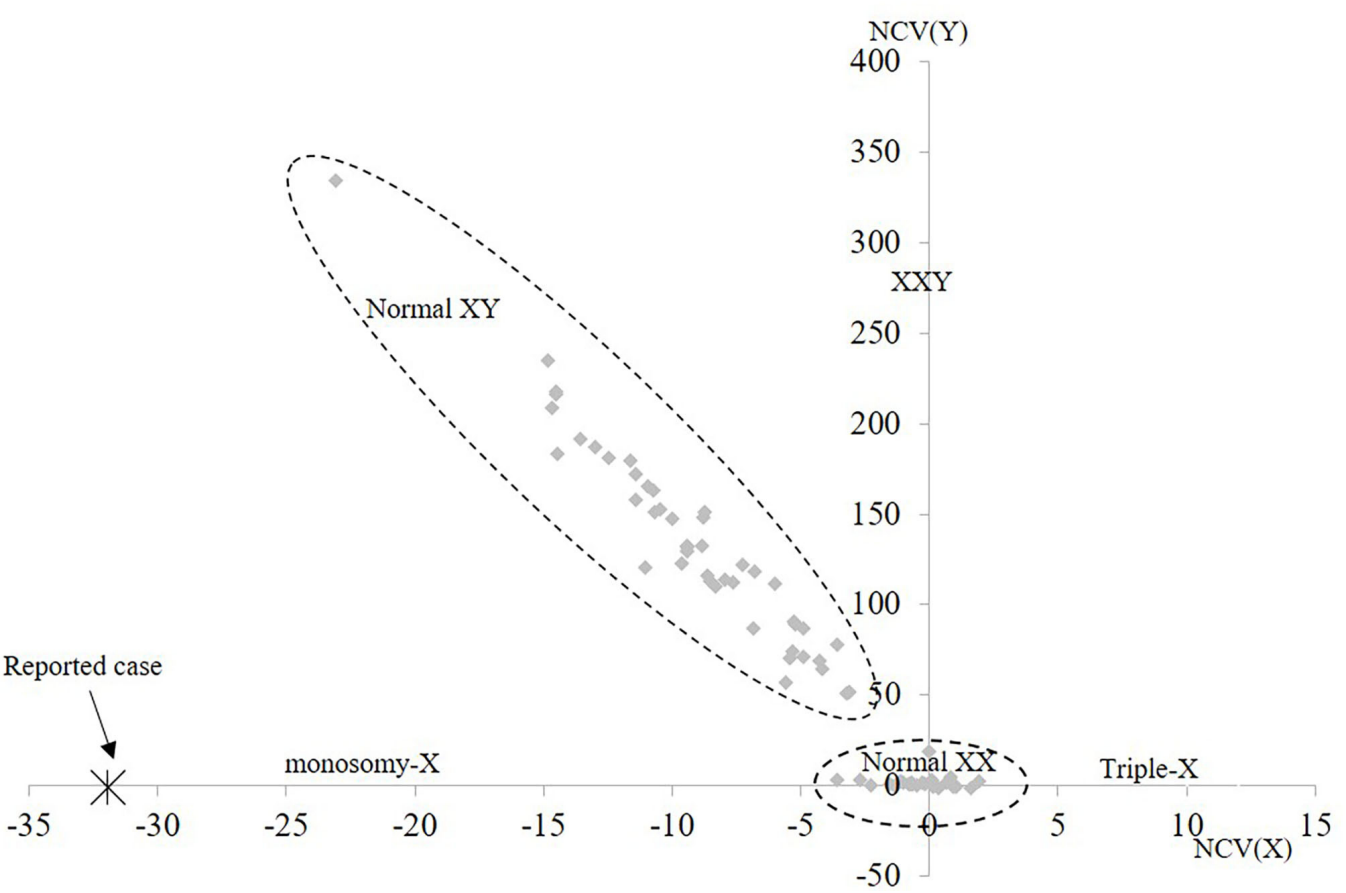
Cell-free NIPT result for chromosome X. The 1st axis represents the normalized level of X-chromosomal material and the 2nd axis represents level of Y-chromosomal material detected. The normal range for female euploid samples (Normal XX) and male euploid samples (Normal XY) are marked by the dotted lines. The cfNIPT result for the reported case is marked with an enlarged X. NCV, Normalized Chromosome Value (for chromosome X and chromosome Y, respectively).

For cfNIPT analysis, the reported cell-free fetal DNA fraction was 7% and the results showed two copies of chromosome 13, 18, and 21. The result was inconclusive for chromosome X, but the decreased proportion of X-chromosomal material was strongly suggestive of Turner syndrome (Figure 1). No Y chromosome material was detected.

**Figure 2:**
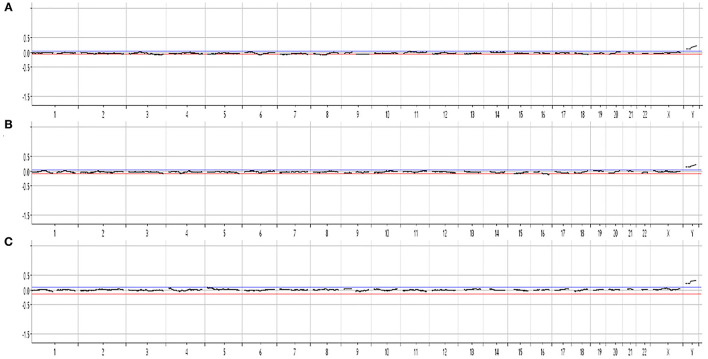
Cell-based NIPT chromosomal microarray of single-cell whole genome amplified DNA from three fetal extravillous trophoblasts **(A–C)**. The result is consistent with a normal female karyotype, arr[GRCh37](1-22,X)x2.

For the first cbNIPT analysis, only one fEVT was isolated, and the STR analysis showed that the sample contained DNA of both fetal and maternal origin, which may occur if both a fetal and a maternal cell is sorted to the same tube by FACS. Hence, no further analysis was conducted on this. In the second cbNIPT, on a new blood sample, fetal origin was validated in three candidate cells and these were analyzed individually by chromosomal microarray. The aCGH results of all three single cell analyses demonstrated no aneuploidy, and two X chromosomes when analyzed against a female control [arr[hg19](1-22,X)x2, Figure 2]. Hence, cbNIPT result was normal and female.

## Discussion

During the last decade, cfNIPT has been introduced worldwide as an efficient screening tool for detection of trisomies 13, 18, and 21 and potentially sex chromosomes, however, the positive predictive value for the SCAs is lower (Gadsbøll et al., 2020; Luo et al., 2021; Pertile et al., 2021). Prenatal diagnosis of SCAs in a fetus is complicated by the absence of confirmatory ultrasound findings beyond the increased nuchal translucency (Christiansen et al., 2016). Further, maternal factors such as SCA mosaicism or age-related loss of the X-chromosome may interfere with data interpretation, hence causing false positive cfNIPT results. Therefore, follow-up invasive prenatal diagnosis is necessary to confirm a positive cfNIPT result, and false positive cfNIPT results for SCA may lead to redundant invasive testing and increased waiting time (Zhang et al., 2020).

In the present case with maternal mosaicism for Turner syndrome, we report discordant results between cfNIPT and cbNIPT. The cfNIPT result was inconclusive for chromosome X, but strongly suggestive of monosomy X, and thus inconclusive. In this case, the cfNIPT result may reflect the mosaic state of Turner syndrome in the pregnant woman alone, or in combination with mosaic or non-mosaic Turner syndrome in the fetus. The cbNIPT was obtained by analysis of three individual fEVTs, and none of the tests showed aneuploidy and all confirmed two X chromosomes in the fetal cells (arr[GRCh37](1-22,X)x2). The cbNIPT result showed normal with female karyotype, but fetal mosaicism is possible and cannot be excluded based on analysis of few fEVTs, however, the likelihood is greatly reduced, and the normal nuchal translucency speaks against this. Unfortunately, follow-up confirmation (chromosome analysis or molecular karyotyping) after birth of the female child has not been obtained for this study. The presented case is a part of a larger study that has been running since 2018, where pregnant women in the Central Denmark Region have been offered cbNIPT in addition to cfNIPT albeit in a research setting (Vestergaard et al., 2017; Hatt et al., 2020).

The cfNIPT analysis offered by the Danish national healthcare system (Veriseq NIPT, Illumina) does not distinguish fetal and maternal cfDNA. Therefore, cfNIPT results may be affected by maternal obesity, maternal copy number variations or mosaicism, abnormal maternal karyotype, as well as confined placental mosaicism, a vanishing twin or malignant tumors (Hui and Bianchi, 2020). In a recent retrospective study, Lund et al. (2021) assessed the early clinical use and performance of cfNIPT in Denmark by collecting cfNIPT data from national public registers and private providers (total *n* = 3,936 NIPT results) from March 2013 to June 2017. SCAs (*n* = 14) constituted 18.4% of the positive cfNIPT results (*n* = 76) and for those with genetic follow-up (*n* = 13), 46% were false positives (*n* = 6). A similar study was conducted by La Verde et al. (2021) on a large cohort of 36,456 consecutive pregnancies in the Italian population tested between April 2017 and September 2019. Of 501 screen-positive cfNIPT results, 28.9% (*n* = 145) were positive for SCA, and of those with follow-up diagnostic testing (*n* = 135), 13.3% (*n* = 18) were false positives, corresponding to a positive predictive value of 86.7% for SCAs. In 11 of the 18 false positive results for fetal SCA, maternal SCA (in a mosaic form) were subsequently identified. Importantly, none of these 11 women conceived without reproductive assistance, which, retrospectively, may be a warning sign against using cfNIPT with the aim of screening for SCA in the fetus. In these cases, cbNIPT could potentially be offered instead of cfNIPT analysis.

Cell-based NIPT is based on analysis of circulating placental cells and is therefore also affected by confined placental mosaicism as are cfNIPT and CVS results. The obstacle for detection of CNVs using cbNIPT is the DNA quality after fixation and staining technique used for enrichment of fEVTs. However, cbNIPT is unaffected by maternal obesity (Kruckow et al., 2019) or maternal mosaicism, and the analysis of a pure fetal genome enables testing for fetal aneuploidies and larger CNVs independent of the maternal chromosomal status. Therefore, cbNIPT may provide a clinically useful NIPT option in cases where maternal factors interfere with the interpretation of cfNIPT results. cbNIPT and new approaches for SNP-based cfNIPT analysis to distinguish fetal- and maternal chromosomal anomalies (Samango-Sprouse et al., 2013) expand the opportunities for non-invasive prenatal testing. Therefore, experiences and pitfalls of different NIPT methodologies, as well as technical advances within the field, must be considered in order to evaluate the effectiveness of the different NIPT strategies and to avoid redundant referral for invasive testing. In conclusion, more data is needed to evaluate the performance of cbNIPT for SCAs in general. However, cbNIPT may potentially provide a non-invasive testing option for aneuploidy screening compatible with maternal SCA or mosaicism as well as age-related loss of the chromosome X.

## Data Availability Statement

The original contributions presented in the study are included in the article/supplementary material, further inquiries can be directed to the corresponding author.

## Ethics Statement

This project has been approved by the Local Danish Scientific Ethical Committee (69335) and the Danish Data Protection Agency (2012-58-006). The pregnant woman gave written informed consent for her case to be published as a case report.

## Author Contributions

LJ, LH, RS, and PS developed the technology for isolation of fetal extravillous trophoblasts. IV managed the project clinically and was responsible for patient's approval, collection of clinical material, and interpretation of clinical results including aCGH and cell-free NIPT along with SM and LA. LJ wrote the first version of the manuscript. All other authors contributed to revisions.

## Funding

Isolation and analysis of fetal extravillous trophoblasts was funded by ARCEDI Biotech. IV's research was funded by a grant from the Novo Nordisk Foundation (NNF16OC0018772) and she receives no funding from ARCEDI Biotech. LJ was doing an Industrial Ph.D funded by ARCEDI Biotech, Aarhus University and an Innovation Fund Denmark grant 0153-00004B.

## Conflict of Interest

LJ, LH, RS, and PS are all employed by the Danish biotech company ARCEDI Biotech that has launched a commercial cell-based non-invasive prenatal test (www.evitatest.com). The remaining authors declare that the research was conducted in the absence of any commercial or financial relationships that could be construed as a potential conflict of interest.

## Publisher's Note

All claims expressed in this article are solely those of the authors and do not necessarily represent those of their affiliated organizations, or those of the publisher, the editors and the reviewers. Any product that may be evaluated in this article, or claim that may be made by its manufacturer, is not guaranteed or endorsed by the publisher.
